# Impact of Ionizing Radiation Exposure on Placental Function and Implications for Fetal Programming

**DOI:** 10.3390/ijms25189862

**Published:** 2024-09-12

**Authors:** Cameron Hourtovenko, Shayen Sreetharan, Sujeenthar Tharmalingam, T. C. Tai

**Affiliations:** 1 Medical Sciences Division, NOSM University, 935 Ramsey Lake Rd., Sudbury, ON P3E 2C6, Canada; chourtovenko1@laurentian.ca (C.H.); shayenthiran.sreetharan@lhsc.on.ca (S.S.); sutharmalingam@nosm.ca (S.T.); 2 School of Natural Sciences, Laurentian University, 935 Ramsey Lake Rd., Sudbury, ON P3E 2C6, Canada; 3 Department of Medical Imaging, London Health Sciences Centre, 339 Windermere Rd., London, ON N6A 5A5, Canada

**Keywords:** fetal programming, ionizing radiation, placenta, growth restriction, antioxidants, reactive oxygen species

## Abstract

Accidental exposure to high-dose radiation while pregnant has shown significant negative effects on the developing fetus. One fetal organ which has been studied is the placenta. The placenta performs all essential functions for fetal development, including nutrition, respiration, waste excretion, endocrine communication, and immunological functions. Improper placental development can lead to complications during pregnancy, as well as the occurrence of intrauterine growth-restricted (IUGR) offspring. IUGR is one of the leading indicators of fetal programming, classified as an improper uterine environment leading to the predisposition of diseases within the offspring. With numerous studies examining fetal programming, there remains a significant gap in understanding the placenta’s role in irradiation-induced fetal programming. This review aims to synthesize current knowledge on how irradiation affects placental function to guide future research directions. This review provides a comprehensive overview of placental biology, including its development, structure, and function, and summarizes the placenta’s role in fetal programming, with a focus on the impact of radiation on placental biology. Taken together, this review demonstrates that fetal radiation exposure causes placental degradation and immune function dysregulation. Given the placenta’s crucial role in fetal development, understanding its impact on irradiation-induced IUGR is essential.

## 1. Introduction

Accidental exposures to high-dose radiation can occur across various sectors, such as the medical, industrial, military, and environmental fields [1,2]. These exposures can pose a significant concern for human health, with pregnant individuals among those of great concern due to the sensitivity of the fetus [3]. With the fetus’s internal organs still developing during gestation, a tighter homeostatic uterine environment is imperative for optimal development [4]. During early gestation, the placenta, an external fetal organ, experiences rapid development to support the environment to which the fetus is exposed [5]. High-dose radiation exposure during pregnancy may cause impairments to placental function, therefore affecting the fetal environment during gestation. Due to the sensitivity of the fetus, some governing bodies, such as the Canadian Government, have reduced the amount of occupational radiation exposure a worker is allowed to be exposed to during the period of pregnancy [6]. Despite the advances in radiation safety protocols, accidents can still occur, necessitating a deeper understanding of the effects of radiation exposure on the placenta. A comprehensive understanding of placental structure is essential to assessing how irradiation can impact the development of the fetus.

## 2. Placental Structure

### 2.1. Mouse Placenta

**Figure 1:**
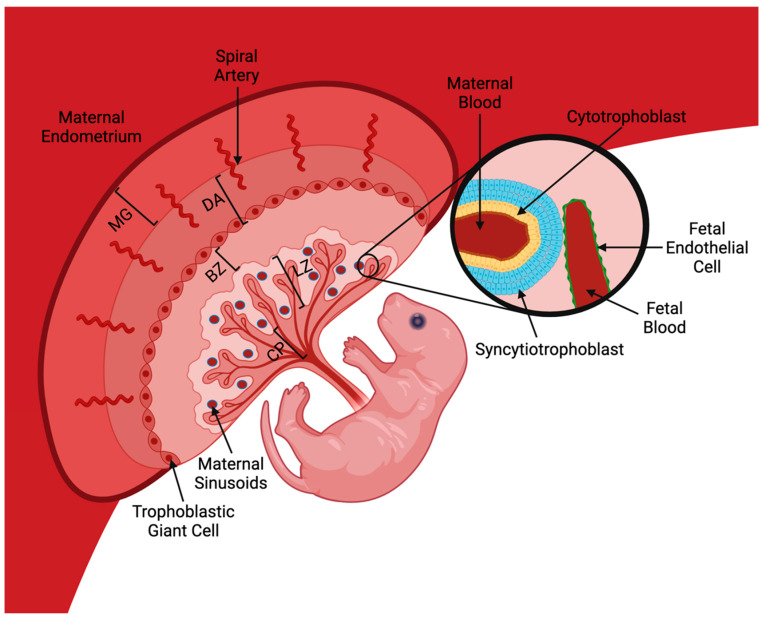
An anatomical diagram of the matured mouse placenta. Metrial gland (MG), decidua (DA), basal zone (BZ), labyrinth zone (LZ), and chorionic plate (CP). Created in BioRender. Hourtovenko, C. (2024) BioRender.com/x27s327 (accessed on 10 September 2023).

Among mammalian species, placental structure can differ depending on the needs of that species during the developmental phase. Mouse placenta can be segmented into five regions: the chorionic plate, labyrinth zone, basal zone, decidua, and the metrial gland (Figure 1) [7]. At the fetal side of the placenta, the chorionic plate is the site where the umbilical cord connects the fetus to the placenta. The chorionic plate comprises two layers of syncytiotrophoblast cells and one layer of cytotrophoblast cells, as seen in Figure 1 [8]. Due to this, rodent placenta is known as a *hemotrichorial* type [7,9,10]. *Hemochorial* refers to the separation of the maternal blood and fetal blood by the chorion, and the *tri* indicates the presence of three cell layers that separate the two [11]. This is important as the separation via trophoblast cells creates a chemical and physical barrier to regulate the uterine environment in which the fetus develops [12]. These cytotrophoblast cells are undifferentiated and are used to help attach the growing fetus to the uterine tissue [13]. In contrast, syncytiotrophoblast cells differentiate from the cytotrophoblasts and are used for nutrient and gas exchange between maternal and fetal blood. Additionally, they act as a barrier, preventing maternal blood from leaking into the uterine cavity [8].

The second structure within the placenta is the labyrinth zone (Figure 1). This area is where maternal blood pools into maternal sinusoids, dilated capillaries, for gas and nutrient exchange. The labyrinth zone consists of protrusions of the chorionic plate [7]. The layer of cytotrophoblast cells comes in direct contact with the maternal blood and contains thousands of microvilli to increase the surface area, improving nutrient and gas transfer. The wall on the other side of the labyrinth is the basal zone (also known as basal plate) (Figure 1), which contains three types of differentiated cells: spongiotrophoblasts, trophoblastic giant cells, and glycogen cells [14]. Glycogen cells form small islands during mid-gestation but develop into larger lacunae during the end of gestation [15]. The lacunae then fill up with glycogen to provide energy during birth. According to Simmons and Cross (2005) [16], the function of spongiotrophoblast cells is not fully understood, but these cells potentially play a role in supporting the structure of the placental vascular system. The trophoblast giant cells play a role in maternal decidual remodelling and hormone secretion to regulate placental development [17]. The chorionic plate, labyrinth zone, and basal plate are crucial as they allow for the transfer of molecules between the maternal and fetal blood.

The decidua and metrial gland consist of both maternal and fetal cells, as this is where the placenta invades the maternal endometrium (Figure 1) [16]. The decidua is the neighbouring structure on the maternal side of the basal plate and initiates the invasion of the fetal placental cells into the maternal mesometrial tissue [18]. The decidua comprises parenchymal decidual cells, immune cells (uterine natural killer cells, macrophages, dendritic cells), and blood vessels [19]. Parenchymal decidual cells are multi-nuclear polyploid cells that produce markers such as prolactin and insulin-like growth factor binding proteins [19,20]. Prolactin plays a role in water and ion transport regulation across the placenta, while insulin-like growth factor proteins play a role in endocrine communication and placental growth [21,22]. Decidualization of the parenchymal cells leads to expansion into the endometrial tissue, forming the metrial gland [18]. The metrial gland comprises decidualized endometrial stromal cells, uterine natural killer cells, spiral-shaped arteries, trophoblasts cells, granulated material gland (GMG) cells, and fibroblasts [12]. The maternal low-flow spiral arteries within the maternal endometrium are converted into high-flow arteries to increase blood flow within the labyrinth zone. The metrial gland, located in the mesometrial triangle, fully develops in mid-gestation but then regresses closer to the end of gestation. The exact functions of the metrial gland are not fully understood, but it does possess some immunological properties due to uterine natural killer cells and granulated metrial gland cells [23]. These two structures are designed to increase blood flow within the labyrinth zone and provide structural support to the rest of the placenta. The various substructures of the mouse placenta are organized precisely for the optimal growth and development of the fetus.

The rodent placenta is known as the *chorioallantoic* placenta [24]. This is important to distinguish as they also have a smaller secondary placenta called the *choriovitelline* placenta, or the inverted yolk sac [25]. The mouse yolk sac fully envelops the embryo and assists gas and nutrient exchange between maternal and fetal blood [26,27]. It consists of a layer of hypoblast cells and a secondary layer of epiblast cells [27]. The yolk sac surrounds the embryo, leaving a pocket of amniotic fluid in which the embryo resides. Despite its role in gas exchange, its importance in fetal development decreases as gestation progresses, due to the *chorioallantoic* placenta taking over in function. However, it is present during the entirety of gestation within mice [26].

### 2.2. Comparison of Human and Mouse Placentas

With the human and mouse placenta being very similar, preclinical mouse studies are performed to help understand human placenta physiology [28]. However, there are some differences which must be noted. The human placenta, also a discoid shape, is hemomonochorial, instead of the hemotrichorial design of the mouse placenta [9]. This means that the maternal blood is only separated from the fetal blood by one layer of trophoblast cells. Diffusion of nutrients and gases through three layers of cells takes more effort and is less efficient. However, it is not the only factor that comes along with nutrient diffusion [28]. Another difference is that the labyrinth zone in the mouse placenta is called the villous membrane in the human placenta [9]. The mouse labyrinth is set up to allow the maternal and fetal capillaries to be arranged parallel to each other, which is ideal for countercurrent exchange. The microvillous structure within the human placenta is an intermediate between countercurrent and parallel flow, which is deemed less efficient at nutrient and gas exchange. During the first half of gestation, the human placenta is a dichorial type, but it develops into the monochorial type in the last half [25]. Another difference is the separation between transport and endocrine function in the labyrinth and junctional areas in mice’s placenta. However, this separation does not occur in the human placenta.

There is also a difference in the number of placental structures between the two. The mouse placenta has a distinguishable separation between the metrial gland and decidua, while the human placenta only consists of the decidua [10]. Despite this, the human decidua has a more significant invasion of the endometrial lining. While the invasion of mouse extravillous trophoblast cells is limited to the decidua basalis, one of the first layers in the uterine wall, human extravillous trophoblasts invade the inner third of the myometrium. There are also a few differences between the choriovitelline placenta [27]. The human choriovitelline placenta is only seen during the earlier stages of gestation, whereas in the mouse it is present throughout gestation [26]. Additionally, the mouse choriovitelline placenta encircles the fetus, whereas it only extends from the abdomen in humans. For a comprehensive review of the human placenta, please refer to the textbook, *Pathology of the Human Placenta* by Benirschke et al. (2012) [9]. Despite the few differences, the similarities allow the mouse placental model to be used within preclinical studies to help improve our understanding of the human placenta.

## 3. Placental Function

### 3.1. Molecule Transfer

During gestation, the internal fetal organs are still developing and not fully functional [29]. Due to this, the placenta has to perform all of the required functions for fetal development, including respiratory, nutrition, excretory, endocrine, barrier, and immunological functions. The structure of the placenta allows for the transfer of nutrients and oxygen from the mother to the fetus without direct fetal/maternal blood contact [7]. The respiration, nutritive aspect, and waste removal occur predominately in the labyrinth zone or villous membrane section of the placenta, depending on whether it is a mouse or human placenta. The endovascular trophoblastic cells dilate the metrial gland uterine spiral arteries to increase blood flow into the labyrinth zone, improving nutrient and oxygen exchange [30]. The labyrinth zone contains syncytial knots to facilitate respiratory function, as it decreases the distance of gas exchange [31]. Glucose, the main carbohydrate transported across the placenta, occurs through the glucose transporter family (GLUT 1, 3, 4, 8, 10, and 12) [32,33]. Due to their smaller size, amino acids can passively diffuse through the trophoblasts [5,34]. Lipids are larger molecules and must be hydrolyzed by lipoprotein lipase and endothelial lipase via the trophoblast cells into nonesterified fatty acids [35,36]. They can then be transported through the placenta via passive diffusion or through various fatty acid transporter proteins. Due to the fetal kidneys and urinary system not being functional during gestation, fetal waste travels back across the labyrinth zone into the maternal blood to be processed and excreted with the maternal waste [5,32].

### 3.2. Endocrine Communication

The placenta is an organ that does not contain any neuronal tissue; communication with the mother and fetus is mediated via the bloodstream, through endocrine communication [5]. The endocrine system consists of maternal, fetal, and placental glands releasing hormones/proteins to assist with fetal development and protection [37]. Examples of factors produced by the placenta are progesterone, estrogen, chorionic gonadotrophin, placental growth hormone, cytokines, and chemokines. The vascular endothelial growth factor is produced by parenchymal decidual cells, which aid in placental growth and the development of blood vessels [38,39]. Cytokines and chemokines are proteins secreted by placental macrophages and dendritic cells for immunological purposes [19]. The chorionic gonadotropin is produced by placental syncytiotrophoblastic cells to stimulate progesterone production to maintain pregnancy [40]. Progesterone and estrogen are sex hormones crucial for fetal organ development, placental function, and fetal protection from the maternal immune system [41]. The placenta does not produce all of the required enzymes for estrogen synthesis, and receives dehydriosoandrosterone sulfate (DHEAS) and dehydriosandrosterone (DHEA) from both the mother and the fetus [37,41]. There have been no studies to our knowledge indicating any maternal hormones that are required for placental function. Since the maternal and fetal blood are separated via trophoblast cells, the placenta also acts as a barrier to help protect the fetus against harmful substances, such as xenobiotics [42]. The trophoblast cells contain many cytochrome P450 enzymes, such as CYP1A1, CYP1B1, CYP2E1, and aromatase, which play a role in the metabolism of xenobiotics [43]. Despite the function of a barrier, some harmful substances can pass through and enter the fetal blood. Lead, arsenic, and other toxic substances have been found to pass through the placental barrier quite easily [44]. Placental transport of molecules can occur either passively or actively through the mechanism of pumps [45].

### 3.3. Barrier and Immunological Properties

The trophoblast layers contain influx/efflux pumps in the syncytiotrophoblast cells of the labyrinth zone, such as multidrug resistance protein 1, a resistance-associated protein family, ATP-binding cassette proteins, etc. [5,45]. The drugs that require active transport are larger than 500 Daltons (Da), while drugs smaller than 500 Da are transported via passive diffusion, facilitated diffusion, phagocytosis, and pinocytosis [45]. Phagocytosis and pinocytosis are the cellular engulfing of large particles and extracellular fluid, respectively [46]. Facilitated diffusion in the placenta is known for transporting antibacterial medication, such as cephalosporins and glucocorticoids, an inflammation-reducing steroid. In addition, maternal antibodies, such as the immunoglobulin G class, can travel through the placenta to the fetus via pinocytosis, providing passive immunity [5]. With granulated material gland (GMG) cells and uterine natural killer cells in the metrial gland, the placenta also provides more immunological properties to help against bacteria and viruses [23].

A thick layer of glycocalyx, a dense gel, covers the microvilli within the intervillous space [9]. It plays a role in preventing pathogens from passing through the trophoblast layers. Viral pathogens tend to be transmitted through the decidual placental interface but have a more difficult time compared to non-viral pathogens, which can cross the placental barrier by cell-to-cell transmission [47]. The dense actin cytoskeleton and a continuous network of syncytiotrophoblast cells with tight cellular junctions make transmission difficult [47,48]. Natural killer cells and macrophages within the decidua reduce the maternal inflammatory response due to the presence of the fetus and protect the fetus from pathogens [48,49]. Dendritic cells and regulatory T cells can also be found within the decidua and play a role in the regulation of the maternal anti-fetal immune response via paternal antigen recognition [50]. In mid-gestation, the transfer of maternal IgG antibodies occurs across the cytotrophoblast/syncytiotrophoblast cell layer in the labyrinth zone [51]. With placental and lactate IgG transfer, passive immunity from the mother can last up to 6 months [52]. The placental immunological function all comes down to the design of its structure.

Cells which undergo extensive damage can initiate apoptosis, programmed cell death, to prevent the resulting damage from continuing to the surrounding cells [53]. Apoptosis is crucial for placental and fetal development; however, exaggerated levels of apoptosis occur within cases of intrauterine growth restriction, hypoxia, and oxidative stress [53,54]. Placental apoptosis is initiated via two pathways, the extrinsic and intrinsic pathways [53]. The extrinsic pathway is initiated by the binding of TNR-R-associated death domains to tumour necrosis factor death receptors, which activates the death-inducing signalling complex. The intrinsic pathway is caused by cellular stress (DNA damage, reactive oxygen species, prions) and results in mitochondrial membrane pore formation. This leads to cytochrome C leaking into the cytosol and initiates the apoptosis terminal pathway [53]. The apoptotic cells are then destroyed via efferocytosis, which is the process of consumption and destruction via macrophages and other phagocytic cells [55]. The lymphatic system is a complex group of vessels and glands which works along with blood vessels to remove and destroy waste and dangerous foreign bodies [56]. The placenta does not contain any lymphatic vessels, so the cytotrophoblast cells stimulate lymphangiogenesis within the uterine wall to help with immune protection and waste removal within the placenta [57]. It is important that the placenta has this mechanism to clear apoptotic and necrotic cells, as they can interfere with placental function, therefore affecting fetal development.

## 4. Placental Development

Following fertilization, while the mouse zygote travels through the fallopian tubes and into the uterus, it undergoes many cell divisions and eventually forms a blastocyte [14]. As the blastocyte implants beneath the uterine epithelial tissue, differentiation of the embryo and placenta begins [58]. This occurs within the first 5 days of gestation [59]. The blastocyte has an outer rim of syncytiotrophoblast (STB) cells, forming a multinucleated structure that creates a protective cavity for the embryoblast. These syncytiotrophoblasts develop into extravillous cytotrophoblast (EVT) cells once the uterine stroma is penetrated. They function in the transformation of the maternal spiral arteries for improved circulation in the labyrinth zone [60]. This invasive activity of the placental trophoblast cells into the maternal myometrium is due to highly active invasion-associated genes, like matrix metallopeptidase [30]. Some extravillous cytotrophoblasts (EVTs) become endovascular trophoblastic cells to transform the already present uterine spiral arteries into wider diameter vessels to allow an increasing blood flow into the labyrinth zone [30,31].

However, during the first half of gestation, these new vessels are plugged by EVT cells to restrict blood flow into the placenta [31]. This creates a low-oxygen stress in the intervillous space necessary for embryogenesis and organogenesis. This low-oxygen environment is crucial during organogenesis due to the fetus’s inability to handle free radicals. This plug is later dissolved when embryogenesis is complete at the end of the first half of gestation [31]. Around gestational day 8, mesoderm cells migrate to the visceral endoderm, developing into vascular cells and primitive vitelline vessels, or the visceral yolk sac [28]. The yolk sac is the first placental structure to arise and becomes visible by gestational day 7.5 [59]. The yolk sac provides nutrients via phagocytic uptake of maternal secretions from the amniotic fluid. Around day 10, other mesoderm cells become allantoid mesenchymal cells and gradually invade the trophoblastic cells already present to form the fetal blood vessels [28]. This is the start of the labyrinth zone [59]. The spiral arteries within the early stages of the decidua develop trophoblast giant cells in place of smooth muscle cells. The shift in nutrition sources from the choriovitelline placental to the chorioallantoic placenta occurs around day 10, with the transition finalizing by day 12.5. From this point forward, the fetus receives most of its nutrients from a countercurrent exchange system via the maternal–fetal vascular system.

The villi at the implantation site become the placenta, and the villi at the opposite side regress, becoming the placental chorionic membrane [31]. Once the villi are entirely developed, the fetal capillary walls come in contact with the outer surface, which results in the formation of the vasculosyncytial membrane (VCM). The VCM, located in the labyrinth zone, is where most of the gas exchange between mother and fetus occurs. Through the formation of syncytial knots from clustering syncytiotrophoblasts, there is a decrease in the distance required for gas exchange. By gestational day 12.5, the chorioallantoic placenta is divided into the maternal decidua, junctional zone, and labyrinth zone [28]. By this time, the placenta reaches its peak in weight, where it plateaus until the regression of the decidua [59]. At this point, 50% of the placental weight is made up of the labyrinth, representing the importance of nutrient and gas exchange for fetal development. In addition, a peak in uterine natural killer cells occurs at this time and decreases until birth. By day 14.5, the cytotrophoblasts will fuse to allow bridges to form, and syncytiotrophoblasts protrude through the bridges to come into contact with the maternal blood, where they release hormones for endocrine communication. The basal zone and the metrial gland are in peak development during mid-gestation, but both start to recede before birth [7]. The degradation and deterioration of the decidua and metrial gland initiate on gestational day 15.5. By day 16, as the decidua reduces its mass, the maternal uterine lining starts to transform back to the structure before implantation. The formation, development, and regression of the placenta over the time of gestation is perfectly timed for the optimal growth and development of the fetus, while allowing for the fetus to detach from the placenta at birth. The development of the human placenta follows a similar pattern as the rodent placenta with the exception of timing, as the human gestational period is 40 weeks. To avoid repetitive information, this manuscript will not delve into human placental development. The reviews by Turco and Moffett (2019) and Benirschke et al. (2012) [9,61] describe each step in immense detail.

## 5. Fetal Programming

The maternal uterine environment and exposure to stressors can create a non-ideal environment (environment outside of homeostasis) for fetal development, also known as fetal programming [62]. Fetal programming is the change in the uterine environment leading to a predisposition to disease later in life, such as diabetes, obesity, hypertension, etc. [63,64,65,66,67,68,69]. The concept of the role of an improper uterine environment in fetal programming was initially studied by Dr. David Barker and labelled as the *thrifty hypothesis* [63,64]. Earlier studies found a correlation between maternal malnutrition and diabetes, along with cardiovascular disease in the offspring. Since then, there has been experimental evidence that different stressors and conditions can result in the programming of the fetus. One crucial manifestation and evidence that fetal programming has occurred is a low birth weight (LBW) and/or intrauterine growth restriction (IUGR) [70,71]. LBW within humans is classified as a full-term birth weight of less than 2500 g, while IUGR can be classified as the birth weight being less than the 10th percentile of the corresponding gestational age to account for preterm births [72,73]. IUGR can occur due to various factors during gestation [74]. Malnutrition, hypoxia, radiation exposure, and smoking during pregnancy have all been shown to result in IUGR [62,75,76,77]. According to Sibley et al. (2005) [78], fetal growth can determine the occurrence of hypertension, metabolic syndrome, diabetes, and heart disease. Fetal size at birth can also affect the survivability of the child during the neonatal stage. It is a very concerning clinical/obstetric outcome, as IUGR offspring have been shown to be at higher risk for diabetes, cardiovascular disease, and obesity.

### 5.1. Nutritional Models

A prominent cause of IUGR that has been intensively studied is the impact of nutrition on animal models [38,63,64,72]. This can include the overconsumption or underconsumption of various nutrients. When maternal food intake was reduced by 30% in Wistar rats, a significant decrease in both the fetal weight and height, as well as in placental weight, was observed [79]. Additionally, a reduction in ATP synthesis despite increased mitochondria biogenesis was found within the placenta. Not only did this indicate fetal underdevelopment but also placental underdevelopment, which may have contributed to the LBW of the fetus. Protein consumption affects fetal programming, as Rodríguez-Trejo et al. (2012) [80] found that a low-protein isocaloric maternal diet during pregnancy in mice resulted in a weight reduction of 30% in the offspring on the seventh postnatal day. They also reported that a maternal low-protein diet (6%) up to 7 days after birth, during the lactation period, led to a decrease in fetal weight of 45% compared to the control group. Ramadan et al. (2013) [81] not only confirmed a significant decrease in fetal weight when the maternal diet was low in protein (5%) but also found that the size of the fetal rat livers decreased by 30%. Another dietary factor which plays a role in fetal programming is maternal fat consumption [82]. A mouse study completed by Campos-Silva et al. (2020) [83] reported that the four-month-old offspring of mothers who consumed a diet of 49% fat during gestation and lactation, compared to the control group of 17% fat, had increased insulin levels and high blood pressure. This may show early signs of insulin resistance in offspring caused by high-fat maternal diets during gestation [84]. Additionally, maternal obesity has been associated with obesity and related complications in children [72]. These studies show the effects of malnutrition and its results on birth weight. Malnutrition leading to low birth weight can result in type 2 diabetes, metabolic syndrome, and cardiovascular disease [64,79].

Malnutrition during gestation can also directly affect the development of the placenta. Pregnant C57BL/6J mice that were undernourished (30% caloric reduction) from gestational days 5.5 to 17.5 had a reduced fetal and placental weight on gestational day 18.5 [85]. The placenta had dysregulation in the expression of fatty acid transporters. A reduction in fatty acid translocase mRNA expression (*fat/cd36*) was found within the male placenta, while *fabp_m_* was increased. The increase in *fabp_m_* may have occurred as a compensatory effect to counteract the reduced *fat/cd36* expression [85,86]. It was determined by H&E staining that the placenta had a 20% reduction in the junctional zone area compared to the normal diet control [85]. The chorionic plate of the placenta also had a significant reduction in proliferating cells via a Ki67 stain, which could explain the reduced junctional zone area. The underdevelopment of the placenta from the undernourished fetuses and the dysregulation of fatty acid transporters may have played a role in the occurrence of IUGR offspring [85]. In another study, pregnant Sprague-Dawley rats exposed to an isocaloric low-protein diet (4% energy from protein compared to 18% in the control group) from gestational day 2 until birth (gestational day 21) had dysregulation in placental amino acid transporters within their MVM [87]. Dysregulation of insulin-AKT signalling was found at a 50% decrease as *akt-thr-308* phosphorylation was observed, with the total protein levels being unchanged. Dysregulation of mTOR protein kinases was also observed within the MVM. The placenta showed a reduction in phosphorylated *S6k1, 4E-BP1,* and *thr37/46* of 61%, 40%, and 42%, respectively. Examining the Na^+^-dependent uptake of ^14^C a-MeAIB for system A amino acid transporter activity and ^3^H-leucine uptake for system L activity, a reduction of 59% and 68% was observed within the MVM, respectively. The maternal low-protein diet resulted in the inhibition of placental insulin signalling, mTOR, and placental amino acid transport, which led to the reduced fetal growth that was observed [87]. Nutrition is an important factor in the development of the fetus. Whether it is due to the mother not consuming a proper diet or placenta underdevelopment reducing nutrient transport to the fetus, a lack of nutrients during gestation can result in the programming of the fetus.

### 5.2. Hypoxic Environments on Fetal Development

The fetus does not only obtain nutrients from the mother but also from oxygen. With the EVT cells blocking the new vessels within the first half of gestation, maternal blood flow is restricted to the villous membrane [31]. This is because the embryo must initially undergo a hypoxic environment, which is crucial for regulating cellular differentiation and tissue repair [76]. However, if the hypoxic state occurs for too long, the fetus will undergo physiological changes in heart rate, arterial blood pressure, and redistribution of cardiac output towards the brain, heart, and adrenals [76,88,89,90]. Although small amounts of fetal hypoxia are essential for cardiogenesis, the formation of the fetal heart, chronic hypoxia can result in changes in gene expression and affect the development of the cardiovascular system [89]. During gestational days 15–21, pregnant Sprague-Dawley rats exposed to a hypoxic environment (10.5% O_2_) exhibited hypomethylation in the CpG islands centered at the transcription start site (TSS) within both fetal and adult offspring [91]. However, the fetal heart had a slight global hypermethylation while the adult offspring had global hypomethylation. Global hypermethylation has been associated with the presence of congenital heart disease in patients [92]. Hypomethylation of *PHLPP-1* found within the fetal heart increases its sensitivity to ischemia/reperfusion injury [91,93,94]. Alterations in the gene coding for protein kinase c epsilon affect its function in infarction prevention, which follows the precondition of ischemia [95]. These will result in smaller birth weights and delay the development of organs, leading to health problems later in life [89].

During the earlier developmental stages of the placenta, a hypoxic environment (2–20% O^2^) is required for trophoblast proliferation and a reduction in differentiation [96,97]. However, an overly hypoxic environment may negatively affect the placental development. A study completed by Yang et al. (2016) [98] exposed mouse trophoblastic stem cells to a hypoxic environment of 0.5% O^2^ for over two days. By day three, there was a 35-fold increase in the level of cleaved-caspase 3 analyzed via Western blot, indicating increased apoptosis. By using a Hoechst 33,342 dye, a 50% increase in trophoblast giant cells was observed, indicating elevated differentiation in the low-oxygen environment. However, these trophoblastic giant cells were smaller in size and expressed lower levels of PRL3D1 compared to normal differentiation. The control group exposed to 20% O^2^ had a 4-fold increase in cell mass after 2 days, while the low-oxygen group did not have any significant cell mass growth after 6 days. This indicates a significant decrease in proliferation caused by the low-oxygen environment [98]. Exposure to an overly hypoxic environment during gestation not only affects the fetal cardiovascular system but also affects the development of the placenta by reducing proliferation and causing apoptosis. To our knowledge, there is no in vivo study which has studied the effects of a low-hypoxic environment on placental structure and function. However, the placenta’s role in fetal programming has been intensively studied.

### 5.3. Role of Placenta in IUGR

The embryonic environment is not solely based on the levels of oxygen, nutrients, and toxins provided by the mother; the transfer of these molecules across the placenta, dictated by its structural development, is also crucial. Abnormalities of the placental villi could be associated with IUGR [99]. An example is straight fetal placental villi with a vascular network of fewer connections, resulting in less blood flow [78]. Additionally, placental villi with more twists and too many connections have been seen within IUGR offspring. This may result in difficulty for blood to flow into the labyrinth and against the chorionic plate. However, this is rare and occurs with placental alterations during late gestation. Underdeveloped spiral arteries were found in IUGR mice after a maternal low-sodium diet [100]. In healthy pregnancies, the uterine arteries converted into low-resistance uteroplacental vessels, but in the presence of IUGR, high-resistance flow velocity wavelengths were found via Doppler velocimetry [100,101,102]. In IUGR, uterine venous samples show high oxygen content with lower amounts of uterine oxygen extraction [78]. IUGR fetuses have also been reported to have lower levels of amino acids (taurine, leucine, and lysine) in their plasma, which means a lower amino acid transfer rate across the placenta. However, this is less dependent on the placental blood flow and more on the microvillous plasma membrane (MVM) [78,103]. Reduced amino acid transport activity across the MVM has been associated with an abnormal pulsatility index and average blood flow velocity in the fetal heart rate and the umbilical artery [78,103,104,105]. MVM lipoprotein lipase, which releases free fatty acids (FFAs), has reduced placental levels in IUGR. Maternal vascular and fetal malperfusion can result in uteroplacental hypoxia [103]. If there is an inadequate invasion of trophoblast cells into the walls of the maternal blood vessels, arthrosis and fibrinoid necrosis of the blood vessels may occur and result in a reduction in the blood flow to the placenta, causing IUGR [102].

Cytotrophoblasts and syncytiotrophoblasts are vital cells within the placenta, as they allow the placenta to anchor on the uterine wall, enabling the transport of nutrients and oxygen without the maternal and fetal blood coming into direct contact with one another [106]. Syncytiotrophoblasts contain small spherical mitochondria with tubular cristae and a less dense matrix, which aids in steroidogenic function. Though syncytiotrophoblast cells are not as good with oxidation compared to cytotrophoblasts, with their more giant mitochondria, they help the fetus adapt slowly to the maternal environment. These cytotrophoblasts have large mitochondria, which are excellent at using oxygen to produce ATP, a significant energy source in the body. The ATP produced from placental mitochondria is the primary source of energy used for placental and fetal growth, transport of hormones and nutrients, and hormone synthesis [107]. With the mitochondrion being a critical organelle for ATP production and the placenta’s ability to transmit nutrients and oxygen to the embryo, Mayeur et al. (2013) [79] examined how mitochondrial function affects placental growth by feeding pregnant mice a diet reduction of 70%. An increase in complex I and IV activity occurred with the addition of increasing mitochondria oxygen consumption. They determined that impairing mitochondrial function results in less fetal and placental development. They suggest that any defect in mitochondrial function may play a role in the effect of malnutrition on the fetus. The placenta’s function in nutrition and respiration relies on the transportation of molecules across the chorion from the maternal and fetal blood. The energy required for this transfer requires ATP production, showing the mitochondria’s importance in fetal development. Toxins can either affect the placenta directly or travel through the placenta and affect the fetus. For example, the maternal consumption of tobacco has been known to result in a lower birth weight but a larger body mass in adulthood [75]. Lead, cobalt, selenium, and other metals can cross the placental barrier [44]. However, the permeability of the placenta to toxins differs depending on the toxin. The placenta does provide the best barrier for cadmium and arsenic [108]. However, this is not a complete barrier, as low concentrations have been recorded passing through the placenta [108]. High levels of cobalt within the placenta led to a reduction in mitochondrial DNA [109]. High levels of lead reduce placental weight when selenium levels are low, and placental manganese levels have a positive relationship with placental size without affecting birth weight. With the placenta performing all of the essential functions for fetal life, dysregulation of placental function can have detrimental effects on fetal development. Improper development of the placental vascular system and mitochondrial activity of the trophoblastic cells can result in growth-restricted offspring. Various stressors have been shown to result in placental underdevelopment, with the commonality of the production of free radicals (such as ROS and RNS) within these stressors [85,98,110].

## 6. Reactive Oxygen Species

Radiation primarily interacts with water, and with cells containing roughly 70% water, it leads to the production of ROS [111,112]. ROS are reactive chemicals that interact with DNA bases, causing single-stranded and double-stranded breaks. ROS produce free radicals, molecules that carry an unpaired valence electron. Oxidative stress is due to large amounts of ROS synthesis compared to elimination [113]. A few examples of ROS are superoxide anion (O_2_^−^), hydrogen peroxide (H_2_O_2_), and the hydroxyl radical (HO) [114]. At low concentrations, ROS can play an essential role in signalling molecules for cells and help with cell protection against microbial attacks and detoxification [114,115,116]. However, ROS at high concentrations can react with proteins, lipids, carbohydrates, and nucleic acids, resulting in irreversible alterations or destruction [117]. ROS are short-lived with only a local effect [115]. With the help of antioxidants, they can be converted into other species, such as from superoxide to hydrogen peroxide. Hydrogen peroxide is considered a non-radical ROS, meaning it is less reactive compared to its superoxide counterpart. This conversion from a free radical to a non-radical ROS occurs in an attempt to bring them to neutral molecules, such as water [118]. ROS has some beneficial properties, such as in killing microorganisms [115]. Low, regulated levels of ROS play roles in processes such as growth factor stimulation, inflammatory responses, cellular differentiation, growth, etc. [119]. High levels of ROS have been associated with cancers, cardiovascular disease, and inflammation [117,120,121].

The NOD-like receptors (NLRs), activated by ROS, play a role in inflammatory response initiation [115]. NLRs are intracellular receptors that pick up on microbial metabolism within the phagocytes and release inflammasomes and inflammatory cytokines, such as IL-1*β* and IL-18 [122]. Genetic mutations within the genes associated with NOD1 and NOD2 receptors have been found in individuals with inflammatory diseases [123]. Links have been found between the hyperactivation of the NLRP3 inflammasome and excess IL-1*β* production within periodic fever syndromes. With NLRs playing a role in inflammatory activation, elevated ROS levels may lead to hyperactivation of the immune system and result in cellular and tissue damage. Reactive oxygen species can be caused by many external stimuli, such as toxic substances (tobacco, alcohol), malnutrition, radiation, etc. [79,113,119,124]. When drugs enter the system, they are metabolized and produce free radicals via three different mechanisms [125]. Free radicals are formed by electron peroxidase oxidation, metabolism and the futile cycling of cytochrome P450s, and the stabilization of CYP2E1 (ROS-generator). In cases of anorexia nervosa, where malnutrition has occurred, increased ROS levels and mitochondrial dysfunction, especially within leukocytes, have been reported [126]. These leukocytes have increased proinflammatory cytokines and ROS production, playing a role in weight loss due to decreased intake and catabolic efficiency on current energy reserves. On the contrary, increased ROS levels are reported with overnutrition, as ROS acts as a secondary messenger for nutrient absorption/digestion [127]. Despite ROS being naturally produced for its role in cell signalling and immune response, tissue and cellular stressors can cause excess levels of ROS, resulting in more harm to the cells/tissues.

On average, a single cell in the human body is exposed daily to 1.5 × 10^5^ interactions with oxidative molecules [128]. Any value larger than this can be considered a high or increased ROS interaction. High levels of ROS can be a factor in disease occurrence, such as cardiovascular disease, neurological disease, cancers, and more [14,129,130]. Increased levels of ROS can cause DNA damage via base modifications and rearrangements, duplications, and activation of oncogenes; this, in turn, can result in carcinogenesis [130,131,132]. High levels of ROS can predispose the mother to preeclampsia when there is a sudden rise in blood pressure due to the poor trophoblastic invasion of the spiral vessels [14]. According to Davis et al. (2015) and Timpka et al. (2016) [133,134], a hypertensive mother during gestation can lead to the improper development of the fetal cardiac tissue, playing a role in future cardiovascular disease in the offspring. Expression of NADPH oxidase (NOX) enzymes within cardiovascular cells is crucial for proper function, and changes in the expression of NOX1, 2, 4, and 5 have been associated with cardiovascular disease [115,135]. The primary function of NOX is to produce ROS [112,119]. Increased ROS levels reduce nitric oxide’s bioavailability, a vasodilator [135]. With ROS playing a role in cellular proliferation and hypertrophy within vascular smooth muscle cells, high levels of ROS may increase vascular resistance [129]. High levels of ROS are also attributed to neurotoxicity due to the hyperactivation of NOX enzymes in microglial cells and other cells such as astrocytes and neurons. However, the mechanism is not fully understood, according to Brieger et al. (2012) [115]. ROS are not only formed by numerous external factors; they are also formed when the mitochondria create ATP for energy [79,106].

Within the mitochondria, NADH and FADH are used to produce electrons along the electron transport chain via oxidative phosphorylation, which results in ATP synthesis [119]. They react with oxygen, an electron acceptor, during the electron transport chain and produce free radicals, such as hydrogen peroxide and superoxide anions. The electrons are transferred to ubiquinone within complexes I and II, then transformed to cytochrome c in complex III, and finally to an oxygen molecule, producing a free radical [119,136]. This change in electron potential energy results in the activation of proton pumps, leading to the synthesis of ATP [137]. Some of these electrons are transferred to O_2_ molecules, resulting in the production of ROS [136]. This low level of ROS production is beneficial for cellular function as it is a byproduct of ATP synthesis [106]. All NOX proteins within the catalytic subunit are C-terminal dehydrogenase domains bound to FAD and NADPH binding sites. According to Brieger et al. (2012) [115], when NOX becomes activated, it transfers electrons to the two heme groups bound at the N-terminal of FAD. Since oxygen binds to the heme groups, it passes two electrons to the oxygen, forming a superoxide anion. NOX1 is mainly found in epithelial cells, while NOX2 is expressed in lower cell levels with a primary function of bacterial defence [119]. NOX3 is highly present in the inner ear and brain and generates superoxide and hydrogen peroxide. NOX4 is mainly located in intracellular organelles of the kidney, blood vessels, fibroblasts, osteoblasts, and neurons. NOX5 is primarily found in lymphoid tissue and testes and produces hydrogen peroxide. Though both NOX4 and NOX5 form hydrogen peroxide, NOX4 is the major source of production [138]. NOX1, 2, and 3 are subunit-dependant for activity, while NOX4 and 5 are constitutively active [115,139]. NOX5 is activated via increased calcium ions within the cytoplasm and within phosphorylation. In summary, NOX is crucial for cell growth and differentiation, and with NOX activation leading to ROS, low levels of ROS are essential for cell viability [140,141].

Reactive nitrogen species (RNS) are another form of free radicals found within the body naturally or made by external stressors [116,142]. The main RNS produced by cells is nitric oxide (NO), which plays a role as a secondary messenger within synaptic transmission, smooth muscle relaxation, etc. NO is produced naturally via several nitric oxide synthases (NOSs) [143]. Activation of these NOSs is reliant on calcium and calmodium. NO also plays a role in the immune system, as it acts as a cytotoxic agent produced by macrophages and is seen in high concentrations within areas of chronic inflammation. Despite the biological benefits of RNS, higher concentrations can be dangerous against the genomic structure. The oxidation of peroxynitrite can nitrate the sugar–phosphate backbone of DNA, and N_2_O_2_ can nitrosate the primary amines of DNA bases, resulting in strand breaks and mutations. Like ROS, natural levels of RNS are necessary for cellular and tissue function. However, elevated concentrations can be harmful [116,142,143].

## 7. ROS in Fetal Programming

Elevated levels of malondialdehyde (MDA), a biomarker of oxidative stress, have been reported in third-trimester fetal plasma with preeclampsia conditions, which have previously been associated with the resulting programming of the fetus [144,145,146,147]. Another indirect biomarker for oxidative stress is thiobarbituric acid reactive species (TBARS), which has been found elevated within maternal plasma undergoing preeclampsia [148,149]. Exposure to various stressors during gestation, such as hypoxia, radiation, and malnutrition, has been associated with fetal oxidative stress [150,151,152]. Five-month-old Sprague-Dawley rats gestationally exposed to intravenous injections of nicotine (2.1 mg/day) via maternally implanted osmotic minipump from gestational day 4 to birth saw increased MDA and superoxide levels and decreased superoxide dismutase (SOD) activity within the aorta [152]. A study by Vega et al. (2015) [153] looked into the effects of gestational malnutrition on oxidative stress within the fetus and adult offspring. Pregnant Wistar rats given a protein-restricted diet (50%) saw elevated MDA and ROS levels within the fetal liver on gestational day 19. Their study seems to indicate sexual differences, as male offspring showed elevated SOD activity, while the females had decreased activity when compared to the control group. When the offspring were allowed to survive until 110 days post-birth, elevated MDA levels within the liver stayed elevated within females; however, they were lower within males. Elevated ROS levels and SOD activity were only found in the males at 110 days post-birth. Both sexes had elevated levels of MDA within their blood plasma. The occurrence of oxidative stress via protein-restricted diets during gestation affected the oxidative stress/antioxidant interaction and varying effects were also found 110 days post-birth [153].

Pregnant C57BL/6J mice irradiated with 50, 300, or 1000 mGy of gamma cell ^37^Cs irradiation on gestational day 15 resulted in a decrease in both the offspring’s body and cardiac weight 16–20 weeks post-birth [154]. It was reported that female cardiac tissue had increased glucose update and glycogen stores at 1000 mGy, while males had a decrease in glycogen stores at 50 and 300 mGy. Within males, there was an increase in antioxidant glutathione peroxidase (GPx) activity at 300 mGy without any change in total ROS levels. However, the females had an increase in SOD activity at 300 and 1000 mGy, as well as a reduction in GPx activity at 1000 mGy. There was a significant reduction in total ROS at the 1000 mGy dose. Additionally, an immune response was present within the offspring’s cardiac tissue, as increased levels of TNF*α* and IL-12p70 were found after 300 mGy, and IL-10 was found elevated within males at 1000 mGy. Gestational radiation exposure resulted in post-natal effects within the offspring’s cardiac tissue, as a reduction in cardiac size, a disruption in the oxidant/antioxidant system, and elevated levels of inflammatory molecules were observed. A human study examining prepubertal children born with a low birth weight found decreased insulin sensitivity and altered oxidant/antioxidant status [155]. Increased levels of MDA and lower levels of vitamin E, an antioxidant, were found within the participants’ blood samples, with the addition of a shorter lag phase [155,156,157,158]. Another study found children between the ages of 8 and 13 who were born small for their gestational day had increased TBARS and lipid peroxidation levels, as well as increased SOD activity of the erythrocytes [159]. Oxidative stress has been found to play a role in the dysfunction of organ systems, such as the kidneys and heart [113,160,161,162]. Elevated levels of ROS during gestation can not only cause complications during pregnancy but can also lead to long-lasting effects on the offspring. Not only can various stressors lead to disruptions in the oxidant/antioxidant balance during gestation but this effect can continue within the offspring throughout adulthood.

## 8. Antioxidants

Cells have many defence mechanisms to reduce the damage by ROS and RNS by converting them into stable molecules. The cell accomplishes this by using antioxidants. Antioxidants can either be endogenous, produced by the cells within the body, or exogenous, requiring consumption through diet [163,164]. For example, manganese superoxide dismutase (MnSOD) increases the dismutation of O_2_^-^ and the formation of H_2_O_2_, which then, catalyzed by glutathione peroxidase and catalase, is converted to oxygen and water [31]. Glutathione peroxidase is an example of an enzymatic antioxidant [165]. There are also non-enzymatic antioxidants, such as vitamin A, which interrupt the free radical chain reactions. Melatonin, also an antioxidant, has been studied in animal models and has shown some evidence for the protection of brain development in the fetus [166]. It accomplishes this by activating glutathione peroxidase and glutathione reductase, which reduce free radicals [167]. Melatonin is effective at oxidative stress protection in the mitochondria, preventing electron leakage within the electron transport chain. Thiol-based antioxidants are great for free radical neutralization and some DNA repair. The positive charge on the thiol-based antioxidant interacts with the negatively charged phosphate backbone, which in turn blocks the reaction of the free radical with the DNA.

Antioxidants not only protect the DNA from oxidation but also have a protective effect on the lipid membrane. Disulfiram, an antioxidant generally used for alcohol abuse, was used in a recent study where it was given to mice before a whole-body irradiation treatment of 4 Gy of ^60^Co-*γ*-rays [168]. A notable decrease in lipid peroxides and microsome peroxidation of 65% was observed in the irradiated compared to the non-irradiated group. The placenta contains various antioxidants, such as manganese superoxide dismutase, glutathione peroxidase, copper superoxide dismutase, and zinc superoxide dismutase [31]. The placental antioxidants are produced by cytotrophoblast and villous stromal cells when exposed to elevated levels of ROS. A study by Jang et al. (2011) [77] showed that hominis placenta hydrolysate, a desiccated placenta, can reduce mouse crypt cellular death caused by 10 Gy of X-ray radiation. Mice injected with 5 and 10 mg kg^−1^ of hominis placenta hydrolysate five days before receiving the irradiation saw a reduction in intestinal crypt cell death of 30.4% and 56.7%, respectively. This protection was evident 24 h after radiation treatment and up to 72 h, when apoptotic cell count was last recorded. The cytotoxicity effect within the crypt cells caused by irradiation is due to increases in ROS production. Furthermore, the hominis placenta hydrolysate may play a role due to the antioxidant effects of the hominis placenta because of the presence of antioxidant collagen peptides and glutathione peroxidase, which is crucial for embryonic protection from oxidative stress [77]. However, endogenous antioxidant production can only adjust to minimal ROS increases, with excess ROS levels being too much to adapt to, resulting in placental oxidative stress. Pregnant Wistar Kyoto rats treated with 0.1 mg/kg per day of dexamethasone (Dex), a stressor which produces ROS, from gestational day 15 to 21 and given 1 mmol/L of TEMPOL (synthetic superoxide dismutase) saw a reduced mean arterial pressure in newborns at 16 weeks of age, from 120 mmHg to 105 mmHg, compared to the group which received the Dex without the TEMPOL [67]. Rats that received 0.1% of EGCG (458 mmol/L) instead of TEMPOL showed a similar decrease in blood pressure. The antioxidants TEMPOL and EGCG also prevented the effects of Dex on offsprings’ body weight, blood pressure, blood plasma catecholamine levels, and adrenal gene expression gene expression associated with catecholamine production (TH and PNMT). Antioxidants are important molecules within the body that neutralize free radicals, preventing damage. Decreasing antioxidants or increasing ROS molecules can result in genomic and tissue damage, possibly leading to the death of the cell.

## 9. ROS on Placenta

According to Rodríguez-Rodríguez et al. (2018) [113], ROS are required for normal embryonic and fetal development and participation in placentation. The fetus and embryo have a low capacity for antioxidants, which makes them more sensitive to elevated ROS levels and potentially causes a reduction in fetal growth. NOX2, which produces ROS, has been known to contribute to a hypercholesterolemia-induced decrease in new blood vessel formation [74]. Since placental blood vessel development is crucial to fetus growth, an increase in NOX2 activity can result in low birth weight (LBW). Accumulating ROS can affect biomacromolecules and lead to increased apoptosis of trophoblast cells, reducing the invasion of these cells into spiral cells and increasing the odds of IUGR [169]. According to Myatt and Cui (2004) [170], the reduction in the trophoblast cell invasion of the spiral vessels has been associated with the incidence of preeclampsia. Hu et al. (2021) [74] performed a histology of blood vessels from the neonatal piglet placenta of a LBW compared to a NBW offspring, and the underdevelopment of the blood vessels was visibly noticeable, with evidence that LBW was associated with placental damage caused by increased ROS levels. They found in LBW neonatal piglets that the number of mRNA for NOX2 was the same as in the NBW placenta, but the number of NOX2 protein was lower, suggesting that the rate of synthesis and degradation of the NOX2 protein is different in LBW compared to NBW piglets. They concluded a reduction in NOX2 expression resulted in the promotion of tube formation and migration in vitro, while overexpression had the opposite effect [74]. A decrease in NOX2 expression results in a higher expression of mRNA VEGF-A, which plays a prominent role in neovascularization [74,169]. The increase in NOX2 expression results in lower VEGF-A mRNA levels [74]. A reduction in VEGF expression correlates with defects in placental angiogenesis [169]. Overexpression of NOX2 affects VEGF-A by decreasing the phosphorylation of STAT3, a transcription factor, which activates VEGF-A [74]. Despite low levels of ROS being crucial for placental development and function, elevated ROS levels reduce placental blood vessel formation and the conversion of maternal low-flow arteries to high-flow spiral arteries [74,170]. This will greatly affect the transfer of nutrients and oxygen across the placenta to the fetus. A stressor which can cause elevated levels of ROS at the cellular level via the indirect mechanism/hydrolysis of water is ionizing radiation.

## 10. Radiation

Ionizing radiation is a type of energy capable of removing electrons from atoms/molecules, leading to the ionization of particles within biological material [171]. Ionizing radiation can come from a multitude of sources, including decaying radioactive isotopes, cosmic rays, and man-made medical devices [111]. Ionizing radiation comes in two forms, electromagnetic (photons), such as X-rays and gamma rays, and particulate (particles), such as alpha and beta particles, as well as neutrons. However, particulate radiation causes more damage to biological tissue than electromagnetic radiation due to the greater density of ionization events, also known as linear energy transfer (LET) [172].

Ionizing radiation can result in two types of chromosomal aberrations [173]. The first type of chromosomal aberrations consists of *unstable* aberrations. These are genomic modifications that lead to the death of dividing cells. Some modifications include large deletions, the formation of dicentrics (chromones with two centromeres), and the formation of ring chromosomes (chromosomes with fused ends). These modifications can cause cell death via two mechanisms: programmed cell death (apoptosis, necrosis, ferroptosis, etc.) due to large levels of damage, and radiation-induced reproductive failure by inhibiting mitosis. The second type of chromosomal aberrations is considered *stable* aberrations and consists of genetic modifications which do not affect cell replication but are passed through cell generations. Some of these modifications include the following: small deletions, reciprocal translocations, and aneuploidy formation [173]. If too many stable aberrations occur, the cells can develop chromosomal instability [174]. This can lead to severe large-scale alterations which can change the expression of thousands of genes and the occurrence of cancers and other diseases within an organism [173,174,175].

The radiation’s total initial kinetic energy per unit of mass of all charged particles is referred to as Kerma [176,177]. This kinetic energy from the air (Air Kerma) is then converted to the tissue (Tissue Kerma) as the energy is transmitted into the tissue. The absorbed dose is the energy deposited into the tissue in the form of ionizations and is approximately 1.1× the value of Air Kerma. The absorbed dose of radiation can be measured in the unit gray (Gy), where 1 Gy is equal to 1 Joule absorbed per kilogram of absorbed tissue [178,179]. The majority of cells that receive an absorbed dose of less than 1 Gy will survive; however, they will undergo chromosomal mutations [180]. Ionizing radiation can affect DNA in two ways. First, the radiation can directly interact with the DNA, resulting in structural changes and the formation of strand breaks within the DNA, namely single-strand or double-strand breaks [171,181]. Secondly, ionizing radiation can affect DNA via a secondary mechanism of action, through the generation of free radicals such as reactive oxygen species (ROS). Radiation exposure during gestation has been shown to lead to various negative effects on the offspring [68,182,183].

## 11. Radiation Exposure and Fetal Programming

Various carrier fields, such as medical workers within the radiological imaging department, aircrew/astronauts, hard-rock miners, nuclear industry workers, and scientists, are at a higher risk of accidental radiation exposure compared to other fields [184]. Radiation exposure during gestation can affect various organ systems within the fetus. Children who were prenatally exposed during the bombings of Nagasaki and Hiroshima had an increased incidence of intellectual disabilities between the ages of 10 and 19 [182]. One study found that exposure in children during gestational weeks 8–15 resulted in a higher incidence of intellectual disability compared to exposure during later weeks [185]. Another study found that children exposed to 0.18 Gy or more radiation during the bombings of Nagasaki and Hiroshima between gestational weeks 8–15 and 16–25 had a significant reduction in school performance between the ages of 6–9 compared to other gestational time points [186]. An approximate 6× increased rate of intellectual disabilities was found following in utero radiation exposure of mothers within 1500 m of the bombings of Nagasaki and Hiroshima compared to offspring from outside the cities [187]. The exposed dose to gamma rays approximately 1000 m from the hypocenters in Nagasaki and Hiroshima is estimated to be around 8 Gy and 3 Gy, respectively [188]. Similar results to those found at Nagasaki and Hiroshima can be found due to the meltdown of Chernobyl, as children 6–7 years of age from Belarus who were gestationally exposed to ^137^Cs and received a mean thyroid dose of 0.39 Gy presented with a lower IQ score (80–89) and the addition of moderate short-term memory and active attention problems [189]. However, those between the ages of 10 and 12 had no significant difference in IQ scores.

C57Bl/6J mice who were gestationally exposed to 1 Gy of X-ray radiation on gestational day 15 and allowed to grow until 17–18 weeks postnatally performed increased rearing during the Social Interaction Task, indicating the occurrence of hyperactivity [190]. No difference was found in the Porsolt Swim Task and the Open Field Task caused by the high dose of irradiation. The same study also had two other mice groups, who received a dose of 50 mGy or 300 mGy. There was minimal dysregulation at 50 mGy and 1 Gy within the brain in genes associated with ROS regulation, synaptic activity, apoptosis, and DNA methylation. However, in the 300 mGy group, there was overall significant dysregulation in multiple brain regions (hippocampus, neocortex, and cerebellum), with a few of the genes being *SOD1* and *2, DNMT1, NOS3,* and *synaptophysin* [190]. An increased incidence of microcephaly, a neurodevelopmental disorder in which the offspring’s head is smaller by at least three standard deviations, was found among children who were pre- and postnatally exposed to radiation from the atomic bombings in World War 2 [191,192]. A study completed by Wood et al. (1967) [187] saw a reduced head size of at least one standard deviation among 17-year-olds who were exposed in utero to radiation from the bombings of Nagasaki and Hiroshima within 1500 m. Fetal ICR mice irradiated with 2 Gy on gestational day 12 and collected 2 h after saw an 80% reduction in relative abnormal spindle-like microcephaly-associated gene (*aspm*) expression within the periventricular region of the brain [193]. *Aspm* is one of the main contributors to the formation of microcephaly, indicating the potential development of microcephaly within the fetal ICR mice if allowed to develop until birth [194]. Radiation exposure during gestation can lead to intellectual disabilities, reduced IQ, hyperactivity, and microcephaly, depending on the dose and the time of exposure during gestation.

Gestational radiation exposure has also been shown the affect the cardiovascular system within offspring. Sreetharan et al. (2019) [68] exposed pregnant C57Bl/6J mice to 5, 10, 50, 100, 300, and 1000 mGy of ^137^Cs gamma radiation on gestational day 15. At 16 weeks of age, the male offspring had a significant reduction in heart rate, while the female offspring did not have any change in heart rate. Both sexes had no change in blood pressure (systolic blood pressure, diastolic blood pre, and mean arterial pressure), but did have a reduced body weight [68]. Another study looked into the cardiac protein levels in 6-month-old and 2-year-old C57Bl/6J mice exposed to 20, 50, 100, and 1000 mGy of X-rays on gestational day 11 [195]. A proteomic analysis dictated dysregulation in 26 (2.7%) and 41 (4.2%) proteins caused by 1000 mGy of radiation 6 months and 2 years after birth, respectively. Out of these downregulated proteins, mitochondrial proteins (*uqcrc1*, *hadh*, etc.) and structural proteins (*tagin2*, *ldb3*, etc.) were significantly downregulated at both time points. Significant alterations of mitochondrial and cytoskeletal protein levels start to appear at 100 mGy. Only 19 proteins (1.6%) were dysregulated by 20 mGy 6 months after exposure. They did not perform a proteomic analysis of 20 mGy at the 2-year timepoint. The upregulation of *hspb6* at both 6 months and 2 years following 1000 mGy may indicate persistent radiation-induced damage to the heart.

Pregnant Sprague-Dawley rats exposed to 1.53 Gy of ^60^Co irradiation on gestational day 18 had the male offspring’s body and organs weighed on days 1, 7, 14, 21, 30, 60, and 91 post-birth [196]. The greatest reduction in body weight occurred at day 30, with the irradiated group weighing 36% less. The liver–body weight ratio was larger on days 7 and 14, then was smaller on days 30 and 91. The kidneys-to-body ratio was smaller on days 14 and 60. The spleens-to-body ratio was larger on days 7 and 60, and smaller on day 30. The thymus-to-body ratio was smaller on day 14 and larger on day 60. Thirty days post-birth not only showed the largest reduction in offspring body weight but also the greatest reduction in organ-to-body ratio, with the exception of the thymus [196]. Another study conducted a laparotomy on Wistar rats where the fetuses were directly exposed to 1.5 Gy of X-ray radiation [197]. The fetuses were either exposed to whole-body radiation, radiation directed to their head and thorax (pelvis and abdomen shielded), or directly to their pelvis and abdomen (head and thorax shielded). The dams were then surgically closed and allowed to give birth in the following days. At 60 days post-birth, the male offspring were placed with the females until 90 days of age or until insemination occurred, where they were euthanized and their testes collected and weighed. All three irradiation groups had a significant reduction in mean testes weight, with the whole body having the greatest effect. A total of 87% of the males who received whole-body irradiation mated with females between days 60 and 90, and 77% of them had positive insemination. Only 68% of the pelvis-only radiated males mated with the female rats, with an 85% positive insemination rate. When compared to the 100% mated and insemination rate of the controls, the radiation exposure to the fetus not only reduced sexual desire within the rats, but difficulty in fertility was observed [197].

The occurrence of cancers in children has been correlated with prenatal radiation exposure. A study by Yoshimoto et al. (1988) [198] investigated the risk of cancer among children who were exposed to prenatal irradiation from the A-bombings of Nagasaki and Hiroshima. Out of the 814 cases examined by the Radiation Effects Research Foundation (RERF), 13 individuals developed cancer, with 6 having an absorbed uterine dose below 100 mGy, 5 between 100 mGy and 1 Gy, and 2 above 1 Gy. The cancers within the offspring affected various organs, including the breast, uterus, bladder, etc. Twelve of the thirteen were diagnosed between the ages of 14 and 39. The remaining case was a 6-year-old female who died of liver cancer and had developed microencephaly and intellectual disabilities following exposure to 1.39 Gy during the first trimester. In contrast, only 5 out of the 816 individuals within the control group developed cancer, with the earliest occurrence being at age 29 [198]. Another study explored the incidence of cancer in the offspring of mothers who worked in the Mayak Nuclear Facility in Oxyorsk, Russia, from 1948 onwards [199]. Among these children exposed to gamma irradiation doses ranging between 3 and 500 mGy, no significance of exposure dose on cancer mortality was found. However, an increased risk of solid cancer mortality was found within an attained age of less than 15, as the excessive relative risk (ERR) per Gy (EER/Gy) value was 50, compared to an EER/Gy value of less than 0 for all other attained ages. The cancer mortality within this younger age group occurred due to liver and eye cancer, and was the result of an in utero exposure of 459.6 mGy and 19 mGy, respectively [199]. Using the data from the Oxford Survey of Childhood Cancers, Stewart et al. (1956) [200] found a 2× increase in the incidence of leukemia, a 5× increase in malignant kidney cancer, and a 2× increase in lymph node cancer among children who were prenatally exposed to maternal abdomen X-rays, resulting in 25+ mGy of irradiation to the fetal gonads. Radiation exposure during gestation can affect various organ systems. It can affect the central nervous system with the occurrences of intellectual disabilities, reduced IQ, hyperactivity, and microcephaly within offspring [182,185,189,190,191,192]. It can also lead to increased HR and affect protein levels associated with mitochondrial activity and cytoskeletal structure within the heart [68,195]. Smaller livers, spleens, thymus, and testes have also been observed following gestational radiation exposure [196,197]. In utero exposure to radiation also affects these organ systems with the increased occurrences of cancer [198,199,200].

## 12. Radiation Effects on the Placenta

### 12.1. In Vitro Model

Radiation effects on the placenta are relatively understudied and represent a gap in our understanding of radiation affecting fetal growth and development. Radiation effects on the placenta have been studied using in vitro cell culture models. A study by Chen et al. (2020) [201] looked at the effects of leptin-modified human umbilical vein endothelial cells (HUVECs) after radiation exposure. A lower dose of radiation to these cells (1.0 or 0.2 Gy) promoted the expression of pro-angiogenic microRNA, which increases the formation of capillary-like tubes, compared to a dose of 20 Gy, which can induce the apoptosis of endothelial cells. This study showed that leptin-induced HUVECs promoted cell proliferation by increasing the production of VEGF-A while also promoting cell migration. Term PHT cells exposed to 0–8 Gy of photon irradiation 24 h post-initial plating had a 73% reduction in human chorionic gonadotropin (hCG) mRNA expression, which is an essential glycoprotein hormone produced by the placenta which promotes the invasion of the cytotrophoblast cells into the uterine lining [65,202]. An increased expression of PARP and cytokeratin 18 indicates increased apoptosis levels [65]. The PHT cells also had an increase in mRNA and protein cyclin-dependent kinase inhibitor 1A levels caused by the irradiation; this is most likely in an attempt to maintain genomic stability. High-dose radiation exposure of placental cells in vitro can induce apoptosis and reduce proliferation [65,201]. However, low doses of irradiation may increase angiogenesis within the placenta [201].

### 12.2. Animal Models

The effects of radiation exposure on the mammalian placenta have also been studied in vivo. Sheep are a model used for placenta studies due to size, ease of handling, short gestational time, and similarity in structure to the human placenta [9,203]. However, other large animals, such as non-human primates and goats, have also been used for placenta studies [203,204]. Finally, rodents are also used to study the placenta due to the availability of gene mutation strains, their large litters, their ease of care, and their short generation time [205]. Naval Medical Research Institute (NMRI) Swiss-type mice that received a whole-body irradiation dose of 2 Gy of X-ray on gestational day 12 displayed reduced fetal and placental weight on gestational day 18 [206]. Kanter et al. (2014) [65] completed a study where pregnant female C57B1/6HNsd mice were subjected to 4 Gy of gamma cell ^137^Cs radiation at a rate of 70 cGy/min on gestational day 13.5. The mice were euthanized on gestational day 17.5 and both fetal and placental weights were recorded. There was a significant decrease in weight for both the placenta and the fetus compared to the sham irradiation control group (0 Gy). The placenta then underwent microarray analysis and a significant increase in the expression of the gene *Vldlr* was reported*. Vldlr* codes for the very low-density lipoprotein receptor protein and has been shown to play a role in inflammation and endoplasmic reticulum function in adipocytes and macrophages [207]. The 4 Gy of radiation received by the pregnant mice may have induced an inflammatory response within the placenta, possibly playing a role in the fetal underdevelopment which occurred [65]. To examine the effects of radiation on the fetus and placenta at different gestational days, Philippe (1975) [208] irradiated Swiss mice on gestational days 9, 10, 11, and 12 with a single whole-body dose of ^60^Co gamma radiation ranging from 0.5 to 5 Gy. A dose of 1.5 Gy or higher on gestational day 9 was lethal to the fetus, with doses less than 1.5 Gy having no effect on the fetus or placenta. A dose of 1 Gy and higher on gestational day 10 resulted in smaller placentas, with a high fetal mortality rate occurring at 1.5 Gy and higher. On gestational day 11, a dose between 0.75 and 1.5 Gy resulted in smaller fetal and placental weight, with fetal malformations and death occurring over 1.5 Gy. On gestational day 12, a reduction in both placental and fetal weight occurred between 1.25 and 4.5 Gy, with fetal malformations starting at 1.5 Gy. The mouse placenta seemed to be the most sensitive to radiation on gestational day 11 [208]. 

Another study aimed to examine whether an irradiation dose specifically targeting the placenta could affect fetal survivability [209]. Pregnant female Wistar rats were put under anesthesia on gestational day 12 and a laparotomy was performed to expose the embryonic site. The rats were split into five groups, a group which received no irradiation, a group that had 4 Gy of X-ray isolated to the placenta, a group that had 4 Gy isolated to the embryo, a group that had 4 Gy that irradiated half of the placenta and half of the embryo, and a group that had 4 Gy to the entire embryo site. Shielding was conducted to allow isolated irradiation. The pregnant rats were then euthanized on gestational day 16 and then fetal weight and survivability were recorded. The group that had 4 Gy of irradiation to the placenta was the only group that did not show a significant decrease in fetal weight compared to the control. There was also a significant difference in the fetal weight between the group that had received the irradiation dose to the placenta and the other treatment groups (only embryo, half placenta/half embryo, and full embryonic site). The placental irradiated group had the lowest mortality rate (3.8%) compared to the full embryonic site, while the half placental/half embryo group had the largest mortality (56.7 and 33.9, respectively). The results of this study show that at gestational day 12, an isolated irradiation dose to the placenta exhibited a weaker effect on fetal development and survival compared to any dose that irradiated the fetus directly [209]. Radiation exposure to the mouse placenta has not only been shown to increase inflammatory molecules but also may be most effective on gestational day 12 [65,208]. However, isolated high-dose radiation exposure to the placenta on this day has a lesser effect on fetal survivability compared to direct fetal exposure [209]. 

### 12.3. Human Studies

There is also limited research available on the effects of radiation exposure within the human placenta. In one study completed by Iversen et al. (1979) [210] where seven pregnant patients diagnosed with genital carcinoma underwent irradiation treatment with a received mid-pelvic dose of 30 Gy, four patients had the placenta completely within the irradiated volume, while the other three had only half of the placenta in the irradiated volume. Five out of the seven patients experienced spontaneous abortion shortly after the irradiation. All seven placentas showed severe degeneration of the villous vessels and minor degeneration of the decidua [210]. Other papers that have been published were on cases where women received an irradiation dose accidentally. Placental analysis of pregnant women from Chechersky, Belorussia, and Pollesky, Ukraine, where the radioactive pollution within the soil was, on average, 10–20 kCi/km^2^ (370–740 MBq/m^2^) due to the meltdown of Chernobyl [109]. Increased placental sizes of 35.7% and 25% were found in the women exposed during their second and third trimesters, respectively. Lymphocyte infiltration was present within the basal zone of the placenta, which may indicate that the enlargement of the placenta is caused by inflammation. Additionally, a reduction in trophoblastic β_2_-globulin and α_2_-microglobulin was found within the placental serum [109]. The two studies that have been completed present conflicting results. The study by Iversen et al. (1979) [210] showed the degeneration of the placenta following exposure to a large irradiation dose. However, Kulakov et al. (1993) [110] did not make clear the average irradiation dose the women received from the radionucleotide pollution. It is possible that they received a much lower dose, which initiated immune-related hypertrophy of the placenta instead of degeneration. However, other studies would have to be conducted to determine if that is the case.

## 13. Conclusions

With the fetus being small and in the early stages of development, it is sensitive to changes in its environment [62]. Changes to the environment can result in the programming of the fetus, leading to a predisposition for diseases throughout its life. One factor that plays a role in the creation of environmental homeostasis throughout gestation is the placenta [7,29]. It performs all of the required functions for fetal life as its internal organs are still developing. Dysfunction of the placenta can result in the programming of the fetus, leading to various health problems [79,99,100]. Many different stressors throughout gestation, such as irradiation, malnutrition, hypoxia, and toxins, can affect the development of the placenta, leading to placental dysfunction. Out of the known stressors that can result in fetal programming, ionizing radiation is the one whose effects on the placenta are understudied and represent an important avenue for future research. The current literature indicates that radiation exposure to the placenta can lead to structural degeneration of the placental villous vessels and decidua, as well as immune dysregulation. The placenta may be most sensitive to whole-body radiation exposure on gestational day 12 [208]. However, other studies would need to be conducted to confirm this.

One aspect on which there is a lack of information is how high-dose radiation exposure at different gestational ages affects placental development. There has only been one study which has examined the change in placental size by various gestational days [208]. However, there has yet to be a study that has investigated an isolated radiation exposure on the placenta (with the fetus shielded) on its development and function following radiation exposure at each gestational day. Histological, transcriptomic, epigenomic, and proteomic analyses of these placentas may provide better insight into how the different developmental stages are affected by irradiation. The placenta produces various antioxidants which are crucial for fetal development [166,167]. However, there is little research on the effects of elevated levels of maternal antioxidant consumption on placental function and its protective measures against radiation exposure. Finally, the majority of the previous literature has used high doses of radiation [211]. However, accidental radiation exposure can involve both low and high doses of radiation. Of the current literature, there have not been any studies conducted to determine the effects of low-dose radiation exposure on placental function [212]. Continuous future studies in these directions will allow us to better understand how accidental radiation exposure affects the developing placenta, and therefore determine its effects on fetal development.
